# SCPRED: Accurate prediction of protein structural class for sequences of twilight-zone similarity with predicting sequences

**DOI:** 10.1186/1471-2105-9-226

**Published:** 2008-05-01

**Authors:** Lukasz Kurgan, Krzysztof Cios, Ke Chen

**Affiliations:** 1 Department of Electrical and Computer Engineering, University of Alberta, ECEFR, 9701 116 Street, Edmonton, AB, T6G 2V4, Canada; 2 Department of Computer Science, Virginia Commonwealth University, 601 West Main Street, Room 204, Richmond, Virginia 23284-3068, USA

## Abstract

**Background:**

Protein structure prediction methods provide accurate results when a homologous protein is predicted, while poorer predictions are obtained in the absence of homologous templates. However, some protein chains that share twilight-zone pairwise identity can form similar folds and thus determining structural similarity without the sequence similarity would be desirable for the structure prediction. The folding type of a protein or its domain is defined as the structural class. Current structural class prediction methods that predict the four structural classes defined in SCOP provide up to 63% accuracy for the datasets in which sequence identity of any pair of sequences belongs to the twilight-zone. We propose SCPRED method that improves prediction accuracy for sequences that share twilight-zone pairwise similarity with sequences used for the prediction.

**Results:**

SCPRED uses a support vector machine classifier that takes several custom-designed features as its input to predict the structural classes. Based on extensive design that considers over 2300 index-, composition- and physicochemical properties-based features along with features based on the predicted secondary structure and content, the classifier's input includes 8 features based on information extracted from the secondary structure predicted with PSI-PRED and one feature computed from the sequence. Tests performed with datasets of 1673 protein chains, in which any pair of sequences shares twilight-zone similarity, show that SCPRED obtains 80.3% accuracy when predicting the four SCOP-defined structural classes, which is superior when compared with over a dozen recent competing methods that are based on support vector machine, logistic regression, and ensemble of classifiers predictors.

**Conclusion:**

The SCPRED can accurately find similar structures for sequences that share low identity with sequence used for the prediction. The high predictive accuracy achieved by SCPRED is attributed to the design of the features, which are capable of separating the structural classes in spite of their low dimensionality. We also demonstrate that the SCPRED's predictions can be successfully used as a post-processing filter to improve performance of modern fold classification methods.

## Background

Protein structures are predicted to provide answers to key questions related to protein function, regulation, and interactions [1,2]. The solved structures are increasingly useful for structure modeling/prediction for unsolved protein sequences that have a closely related (similar) sequence with a known structure [3,4]. Homology modeling, one of the most successful paradigms used to predict the structure, is based on the assumption that similar sequences share similar folding patterns [5,6]. Sequence alignment which allows for finding similar sequences among the known structures [7,8] usually does not perform well when no sequences with high identity are available. At the same time, structurally similar proteins that share low sequence identity with the sequences used for prediction can be found based on coarse grained classifications such as those provided in Structural Classification of Proteins (SCOP) database [9,10]. This database implements a hierarchy of relations between known protein and protein domain structures, in which the first level is known as the structural class. Prediction of structural classes is based on identifying folding patterns based on thousands of already categorized proteins and using these patterns for millions of proteins with unknown structures but known amino acid (AA) sequences. There are four major structural classes: all-α, all-β, α/β, and α+β. The all-α and all-β classes represent structures that consist of mainly α-helices and β-strands, respectively. The α/β and α+β classes contain both α-helices and β-strands which are mainly interspersed and segregated, respectively [9]. SCOP also defines three additional classes, i.e., multi-domain, membrane and cell surface, and small proteins, as well as four supplementary categories, i.e., coiled coil, designed, and low resolution proteins and peptides. The proposed method targets the four main classes due to two factors: (1) about 90% of SCOP entries belong to the four classes, and most of the existing structural class prediction methods also target these classes [11]. At the same time, the growing number of proteins that are categorized into the other classes motivates development of the corresponding predictive methodologies. We note that the CATH (Class, Architecture, Topology and Homologous superfamily) database [12] defines three main structural classes: mainly-α, mainly-β, and mixed (the fourth class includes irregular proteins that are composed mostly of coils), which approximately correspond to the all-α, all-β, and combination of the α/β and α+β classes in SCOP. We address the SCOP based classification, as it further subdivides the mixed proteins and since most of the existing structural class prediction methods are also based on this definition of the structural classes. Currently, the structural classes in SCOP are assigned manually based on the known structures, while in the past several automated assignment methods were proposed. They include a method proposed by Chou [13] and another by Eisenhaber and colleagues [14], see Table 1. We note that the first assignment method requires knowledge of structure (to distinguish between parallel and antiparallel sheets) and the second one is based purely on the content of the two secondary structures and merges α/β and α+β classes into a mixed class. At the same time, the assignment performed in the SCOP database is more complex and governed by different rules for the α/β and α+β classes. The classification of protein structures in SCOP is performed manually by experts and is based on evolutionary relationships and on the principles that govern their three-dimensional structure [9]. The structural classes are defined based on grouping of the assigned folds, which in turn are categorized based on similarities in spatial arrangement of the protein structure. The folds are assigned to the classes on the basis of the secondary structures, in terms of both their content and spatial arrangement, of which they are composed. In case of all-α and all-β classes they include folds composed mostly of α-helices and β-sheets, respectively. The α+β class includes folds in which α-helices and β-strands that are largely interspersed, while the in case of α/β class which are segregated [9]. Therefore, the assignment into the latter two classes requires the knowledge of the spatial arrangement of the α-helices and β-strands. Since this manual procedure cannot be directly traced using the input sequence or even its corresponding secondary sequence, a variety of methods that predict the structural class based on the protein sequence were developed to facilitate automated, high-throughput assignment. We note that the manual assignment of structural classes in SCOP does not use the features or model applied in the proposed method, as the SCOP assignment is based on spatial arrangement of secondary structure segments, while our method is based on the flat secondary structure sequence.

**Table 1:** Rules for assignment of structural classes based on the content of the corresponding secondary structures.

reference	structural class	α-helix amount	β-strand amount	additional constrains
[13]	all-α	≥ 40%	≤ 5%	
	all-β	≤ 5%	≥ 40%	
	α+β	≥ 15%	≥ 15%	more than 60% antiparallel β-sheets
	α/β	≥ 15%	≥ 15%	more than 60% parallel β-sheets

[14]	all-α	> 15%	< 10%	
	all-β	< 15%	> 10%	
	mixed	> 15%	> 10%	

Prediction of the structural classes is performed in two steps: 1) the AA sequences are transformed into a fixed-length feature vectors; 2) the feature vectors are fed into a classification algorithm to generate a prediction outcome. Numerous in-silico structural class prediction methods were developed. Majority of them use relatively simple features such as composition vector, auto-correlation functions based on non-bonded residue energy, polypeptide composition, pseudo AA composition [15] and complexity measure factor [13,16-25]. Several recent methods use more advanced feature vectors that either combine physicochemical properties and sequence composition, or optimize a selected type of the features [26-29]. Predictions are performed using a wide range of classification algorithms such as fuzzy clustering [30], neural network [31], Bayesian classification [32], rough sets [33], component-coupled [18-20], information discrepancy [22-24], logistic regression [26-29], decision tree [23,34], and support vector machine [27,34-36]. In recent works complex classification models such as ensembles [27], bagging [34], and boosting [22,37] were explored. Unfortunately, some of these methods were tested on small datasets, often with relatively high sequence identity, which resulted in high prediction accuracy [26]. A recent review by Chou provides further details and motivation for development of structural class prediction methods [11]. A feasible alternative for above methods is to use the predicted secondary structure, which can be obtained with accuracy of over 80% for highly similar sequences [38], to assign the corresponding structural classes, e.g., by using one of the abovementioned assignment methods. The main drawback is that in this case the prediction would concern only the all-α, all-β and mixed (which combines α/β and α+β classes) classes.

Development of high quality prediction methods for sequences that are characterized by low identity with sequence used to the prediction continues to be a challenging task. Majority of current secondary structure prediction methods use sequence alignment that requires at least ~30% identity between the query sequence and sequence(s) used to predict its structure [39]. More than 95% of protein chains characterized by a lower, 20–25%, pairwise identity, also referred as the twilight-zone similarity, have different structures [40], which substantially reduces accuracy of the corresponding predictions. For instance, recent research shows that the accuracy of the secondary structure prediction methods trained and tested on protein set in which any pair of sequences shares twilight-zone similarity drops to 65–68% [41]. Similarly, although structural class prediction accuracies for datasets in which training and test sequences share high pairwise sequence identity reach over 90%, they drop to 57–63% when training and testing is performed using datasets in which any pair of sequences has twilight-zone similarity [26,29,32]. At the same time, about 40% of sequences for which the tertiary structure was deposited to Protein Data Bank (PDB) in 2005 share twilight-zone pairwise similarity with any sequence deposited in the PDB before 2005 [29], which motivates development of the prediction methods for these challenging sequences. Most importantly, pairs of sequences with low identity can share similar folding patterns or overall structure [42,43] and can be used to predict tertiary structure [44]. Research also shows that finding similar folding patterns among the proteins characterized by low sequence identity is beneficial for reconstruction of the tertiary structure [45,46].

Large number of proteins chains that are of interest to the biologists (which are being deposited to PDB) and that share twilight-zone pairwise identity with the chains for which the structure is known, and the potential structural similarities between these protein sequences that can be exploited to build more accurate structure prediction methods serve as our motivation. One solution to improve predictions for sequences that share twilight-zone pairwise identity with sequences used to perform predictions is to use a large library of reference functional sequence motifs to build a feature vector that can provide higher accuracy. Such method that uses 7785 features was proposed in [47]. Our goal is to introduce a novel in-silico method that uses a compact and intuitive feature vector to provide accurate prediction of the structural classes for the sequences that have twilight-zone pairwise identity with the sequences used to perform predictions, which in turn could be used to find structurally similar protein that share low sequence similarity.

The proposed method, named SCPRED, uses a custom-designed feature vector that includes 9 features and a support vector machine classifier to generate predictions. Our method is based on the fact that the structural classes are defined based on the secondary structure, although we note that the assignment in SCOP is based on the spatial arrangement of the secondary structure, while our method uses only the secondary structure sequence. We use the secondary structure predicted from the protein sequence by the PSI-PRED [48,49] to develop a novel set of successful features that allow accurately classifying all four structural classes. These features together with a comprehensive set of features used in prior research are used to carefully design, by using feature selection, a compact and well performing feature vector. We also demonstrate that SCPRED can be applied to improve performance of other related prediction methods. Our tests show that coupling of the proposed method as a post-processing filter with state-of-the-art fold classification methods such as PFP [50] and PFRES [51] improves their performance.

## Results and discussion

The experimental evaluation was performed using 10-fold cross validation and jackknife tests to avoid overfitting and assure statistical validity of the results [17,26,52]. The tests were performed on the 25PDB dataset, which includes 1673 sequences which share twilight-zone pairwise similarity, i.e., any pairs of sequence in this set shares twilight-zone similarity. We also use another low-identity dataset, FC699, to evaluate value added of using SCPRED's predictions to improve accuracy of protein fold predictions performed with PFP and PFRES methods. The reported results include the overall accuracy (the number of correct predictions divided by the total number of test sequences), accuracy for each structural class (number of correct predictions for a given class divided by the number of sequences from this class), Matthews's correlation coefficient (MCC) for each structural class, and generalized squared correlation (GC^2^). The MCC values range between -1 and 1, where 0 represents random correlation, and bigger positive (negative) values indicate better (lower) prediction quality for a given class. Since MCC works only for binary classification, we also reported GC^2^, which is based on χ^2 ^statistics. The GC^2 ^values range between 0 and 1, where 0 corresponds to the worst classification (no correct predictions) and 1 corresponds to perfect classification. MCC and GC^2 ^are described in [53].

We note that current secondary structure prediction methods achieve the average accuracy close to 80%, e.g., EVA server reports that PSI-PRED provides the average accuracy of 77.9% for 224 proteins (tested between Apr 2001 and Sept 2005) [54]. Since the average accuracy of PSI-PRED predictions was 77.9% and 77.5% for the 25PDB and FC699 datasets, respectively, we believe that the presented results provide a reliable estimate of the future performance of the proposed method.

### Comparison with structural class prediction methods

The SCPRED was comprehensively compared with over a dozen of competing structural class methods which use various feature vectors and classifiers. The comparison includes three groups of modern methods:

- methods that apply optimized feature vectors [26-28],

- advanced multi-classifier methods including boosting [23], ensembles [27], and bagging [34],

- methods that use the best performing SVM [36] and information discrepancy based classifiers [22,24].

Classification results for the competing methods and the SCPRED are compared in Table 2. The SCPRED, which uses only 9 features, obtained 80% accuracy for both out-of-sample tests. The second best method, which was also designed using 25PDB dataset (in which training and test sequence share twilight-zone identity) [29], obtained 63% accuracy. The remaining, competing methods obtain accuracies that range between 35% and 60%. The relatively low accuracies obtained by the competing methods are due to using a challenging 25PDB dataset [29]. We note that some of these methods [22-24,26,32,34] were originally testes on datasets characterized by higher sequence similarity, which resulted in higher reported accuracies. The methods that reach 60% accuracy are based on a custom-designed feature vectors that includes sequence composition and physicochemical properties [27]. We observe that the usage of simple, composition-based features results in lower accuracy. The results also show that the SVM and logistic regression classifiers perform well on this challenging problem.

**Table 2:** Experimental comparison between SCPRED and competing structural class prediction methods.

Test type	Algorithm	Feature vector (# features)	Reference	Accuracy					MCC				GC^2^
					
				all-α	all-β	α/β	α+β	overall	all-α	all-β	α/β	α+β	
Jackknife	SVM (Gaussian kernel)	CV (20)	[36]	68.6	59.6	59.8	28.6	**53.9**	0.52	0.42	0.43	0.15	**0.17**
	LogicBoost with decision tree	CV (20)	[23]	56.9	51.5	45.4	30.2	**46.0**	0.41	0.32	0.32	0.06	**0.10**
	Bagging with random tree	CV (20)	[34]	58.7	47.0	35.5	24.7	**41.8**	0.33	0.26	0.22	0.06	**0.06**
	LogitBoost with decision stump	CV (20)		62.8	52.6	50.0	32.4	**49.4**	0.49	0.35	0.34	0.11	**0.13**
	SVM (3^rd ^order polyn. kernel)	CV (20)		61.2	53.5	57.2	27.7	**49.5**	0.46	0.35	0.39	0.11	**0.13**
	Multinomial logistic regression	custom dipeptides (16)	[28]	56.2	44.5	41.3	18.8	**40.2**	0.23	0.20	0.31	0.06	**0.05**
	Information discrepancy^1^	dipeptides (400)	[22, 24]	59.6	54.2	47.1	23.5	**47.0**	0.46	0.40	0.24	0.04	**0.12**
	Information discrepancy^1^	tripeptides (8000)		45.8	48.5	51.7	32.5	**44.7**	0.39	0.39	0.25	0.06	**0.11**
	Multinomial logistic regression	custom (34)	[27]	71.1	65.3	66.5	37.3	**60.0**	0.61	0.51	0.51	0.22	**0.25**
	SVM with RBF kernel	custom (34)		69.7	62.1	67.1	39.3	**59.5**	0.60	0.50	0.53	0.21	**0.25**
	StackingC ensemble	custom (34)		74.6	67.9	70.2	32.4	**61.3**	0.62	0.53	0.55	0.22	**0.26**
	Multinomial logistic regression	custom (66)	[26]	69.1	61.6	60.1	38.3	**57.1**	0.56	0.44	0.48	0.21	**0.21**
	SVM (1^st ^order polyn. kernel)	autocorrelation (30)		50.1	49.4	28.8	29.5	**34.2**	0.16	0.16	0.05	0.05	**0.02**
	SVM (1^st ^order polyn. kernel)	custom (58)	[29]	77.4	66.4	61.3	45.4	**62.7**	0.65	0.54	0.55	0.27	**0.28**
	Linear logistic regression	custom (58)		75.2	67.5	62.1	44.0	**62.2**	0.63	0.54	0.54	0.27	**0.27**
	SVM (Gaussian kernel)	PSI-PRED based (13)	this paper	92.6	79.8	74.9	69.0	**79.3**	0.87	0.79	0.68	0.55	**0.55**
	SVM (Gaussian kernel)	custom (8 PSI-PRED based)	this paper	92.6	80.6	73.4	68.5	**79.1**	0.87	0.79	0.67	0.54	**0.54**
	**SCPRED**	custom (9)	this paper	92.6	80.1	74.0	71.0	**79.7**	0.87	0.79	0.69	0.57	**0.55**

10-fold cross validation	SVM (Gaussian kernel)	CV (20)	[36]	67.9	59.1	58.1	27.7	**53.0**	0.51	0.42	0.41	0.14	**0.16**
	LogicBoost with decision tree	CV (20)	[23]	51.9	53.7	46.5	32.4	**46.1**	0.38	0.37	0.31	0.07	**0.10**
	Bagging with random tree	CV (20)	[34]	53.5	51.0	37.6	22.0	**41.2**	0.28	0.30	0.22	0.04	**0.06**
	LogitBoost with decision stump	CV (20)		63.2	53.5	50.9	32.4	**50.0**	0.48	0.36	0.36	0.12	**0.14**
	SVM (3^rd ^order polyn. kernel)	CV (20)		61.4	54.0	55.2	27.4	**49.2**	0.46	0.35	0.37	0.10	**0.13**
	Multinomial logistic regression	custom dipeptides (16)	[28]	56.9	44.2	42.2	17.7	**40.2**	0.24	0.20	0.32	0.04	**0.06**
	Multinomial logistic regression	custom (34)	[27]	69.9	65.3	66.5	38.4	**60.0**	0.60	0.52	0.51	0.23	**0.25**
	SVM with RBF kernel	custom (34)		70.2	61.6	67.6	39.6	**59.8**	0.60	0.49	0.53	0.22	**0.25**
	StackingC ensemble	custom (34)		73.4	67.3	69.1	29.8	**59.9**	0.59	0.52	0.54	0.18	**0.25**
	Multinomial logistic regression	custom (66)	[26]	69.1	60.5	59.5	38.1	**56.7**	0.56	0.44	0.48	0.20	**0.21**
	SVM (1^st ^order polyn. kernel)	autocorrelation (30)		52.4	49.7	0.3	30.4	**35.1**	0.18	0.16	0.05	0.06	**0.02**
	SVM (1^st ^order polyn. kernel)	custom (58)	[29]	77.7	66.8	60.7	45.4	**62.8**	0.64	0.54	0.54	0.28	**0.28**
	Linear logistic regression	custom (58)		74.7	66.4	62.7	45.8	**62.4**	0.63	0.54	0.54	0.27	**0.28**
	SVM (Gaussian kernel)	PSI-PRED based (13)	this paper	93.2	79.5	75.7	69.4	**79.7**	0.87	0.79	0.70	0.55	**0.55**
	SVM (Gaussian kernel)	custom (8 PSI-PRED based)	this paper	92.5	80.4	73.7	68.0	**79.0**	0.87	0.79	0.67	0.54	**0.54**
	**SCPRED**	custom (9)	this paper	92.8	80.6	74.3	71.4	**80.1**	0.87	0.79	0.70	0.57	**0.56**

^1^This method was not originally tested using 10-fold cross validation and thus we also did not report these results

The most accurate predictions are obtained for the all-α class (nearly 92% accuracy), while the best results for the all-β and α/β classes are 81% and 75%, respectively. 70% accuracy is obtained for the α+β class. Similar trend is observed for all tested methods although the corresponding accuracies are lower. The main reason for good performance for the all-α class is that these sequences are α-helix rich and the helical structures are the easiest to predict, i.e., a helix consists of a single segment and is characterized by a repetitive structure.

Table 2 also shows prediction results where the same SVM classifier as in the proposed SCPRED method is applied, but only using the features based on the secondary structure predicted with PSI-PRED ("SVM (Gaussian kernel); PSI-PRED based (13)" rows in Table 2). In this case, the input vector for SVM includes 13 features. We observe that SCPRED that uses features based on sequence and secondary structure is characterized by a smaller feature set and slightly higher prediction accuracy, i.e., the improvement equals 0.4%. The differences are small, and they clearly indicate that the primary source of the information that assures the accurate predictions is the secondary structure predicted with PSI-PRED.

We also performed an experiment where only the 8 PSI-PRED based features from the sequence representation used by SCPRED were used for the prediction ("SVM (Gaussian kernel); custom (8 PSI-PRED based)" rows in Table 2). In this case, the prediction accuracy deteriorated by less than 1% when compared with SCPRED, which again confirms that predicted secondary structure provides the bulk of useful information for the proposed prediction method. The main difference concerns α+β class where SCPRED obtains better results due to the use of the *CV*_*L*---*G *_feature (see Analysis of the Proposed Feature Vector section for more details).

The results show that the proposed feature vector results in significantly improved ability of the classifier to separate structural classes and that SCPRED method provides better predictions when compared with modern, competing methods.

### Comparison with predictions based on secondary structure predicted with PSI-PRED

Since the SCPRED's predictions use the predicted secondary structure, we also compared our method with the assignment methods that are based on the secondary structure. We note that the assignment method by Chou [13] requires knowledge of the tertiary structure to differentiate between α/β and α+β classes, and the method by Eisenhaber and colleagues [14] combines these two classes into mixed class. Therefore, the assignment was performed assuming only three structural classes: the all-α, all-β and mixed class (α/β and α+β classes combined). The two assignment methods were applied with the PSI-PRED predicted secondary structure, which is also used to compute features of the proposed SCPRED method. The corresponding predictions on the 25PDB dataset are compared in Table 3. Since the assignment methods only use the predicted secondary structure, i.e., there is no model to train, they do not require out-of-sample testing.

**Table 3:** Experimental comparison between SCPRED and structural class assignment methods based on the secondary structure predicted with PSI-PRED.

Prediction/assignment method	Accuracy			
	
	all-α	all-β	mixed	overall
[13]	78.8	30.2	66.7	**60.3**
[14]	91.6	73.1	86.8	**84.5**
SCPRED (10-fold cross validation)	92.8	80.6	89.2	**87.9**
SCPRED (jackknife)	92.6	80.1	88.9	**87.6**

The results show that the SCPRED provides more accurate predictions, i.e., 15.5% error rate of the more accurate assignment proposed by Eisenhaber and colleagues was reduced by 3.4/15.5 = 21% in case of using SCPRED. This corresponds to 260 incorrect predictions for the automated assignment, while SCPRED made only 203 mistakes. At the same time, SCPRED is capable of predicting α/β and α+β classes while the automated assignment combines these two classes together.

### Analysis of the proposed feature vector

The proposed vector uses 8 features based on the secondary structure predicted with PSI-PRED, and one based on collocation of Leucine and Glycine (see Materials and Methods for details). Each feature was further analyzed to focus our discussion on the most significant features. We performed prediction on 25PDB dataset using each feature individually and using all but one feature at the time, see Table 4. The removal of individual features results in prediction accuracies that are relatively similar to the accuracy when using all 9 features. The corresponding degradation of the accuracy ranges between 0.5% (when excluding PSIPRED-*CMV*_*H*_^1 ^feature) and 1.4% (when excluding PSIPRED-*NAvgSeg*_*E *_feature) showing that the remaining features still provide good quality predictions. The results obtained when using individual features show that PSIPRED-*NCount*_*H*_^8 ^and PSIPRED-*CV*_*E *_features provide the highest overall accuracy and are among the top two features with respect to prediction of all-β and α+β, and all-α and α/β classes, respectively. They also describe different secondary structures and as such are complementary to each other.

**Table 4:** Comparison of accuracy when predicting the structural classes using all features, each feature individually, and when excluding one features at the time.

Features		Accuracy when predicting with one feature				
		
		all-α	all-β	α/β	α+β	overall
All features included		92.8	80.6	74.3	71.4	80.1

using only one feature	PSIPRED-*NCount*_*H*_^6^	**89.6**	58.7	32.9	46.7	58.4
	**PSIPRED-*NCount***_*H*_^8^	81.9	**78.3**	53.8	**58.7**	**69.0**
	PSIPRED-*CMV*_*H*_^1^	76.3	74.5	**55.8**	48.8	64.3
	PSIPRED-*NAvgSeg*_*H*_	49.9	**83.3**	0.0	47.8	47.9
	PSIPRED-*NCount*_*E*_^5^	85.8	59.1	50.9	47.4	61.4
	**PSIPRED-*CV***_*E*_	**88.9**	71.3	**60.7**	51.0	**68.4**
	PSIPRED-*MaxSeg*_*E*_	83.1	48.8	0.0	**67.1**	52.6
	PSIPRED-*NAvgSeg*_*E*_	79.2	33.9	3.2	42.4	41.8
	***CV***_*L*---*G*_	73.8	0.0	54.3	7.7	32.8

excluding the listed feature	PSIPRED-*NCount*_*H*_^6^	92.1	79.5	71.7	70.8	78.9
	PSIPRED-*NCount*_*H*_^8^	93.0	79.5	73.1	70.3	79.3
	PSIPRED-*CMV*_*H*_^1^	92.5	80.8	72.5	71.0	79.6
	PSIPRED-*NAvgSeg*_*H*_	92.5	81.0	71.4	68.7	78.8
	PSIPRED-*NCount*_*E*_^5^	90.7	80.1	73.4	71.4	79.3
	PSIPRED-*CV*_*E*_	91.9	80.6	72.5	71.4	79.5
	PSIPRED-*MaxSeg*_*E*_	92.8	79.7	73.1	69.8	79.2
	PSIPRED-*NAvgSeg*_*E*_	92.3	80.6	71.1	69.2	78.7
	*CV*_*L*---*G*_	92.8	80.6	73.4	68.9	79.3

Bold font shows the top two highest accuracies when using individual features, and features selected for further analysis.

**Figure 1:**
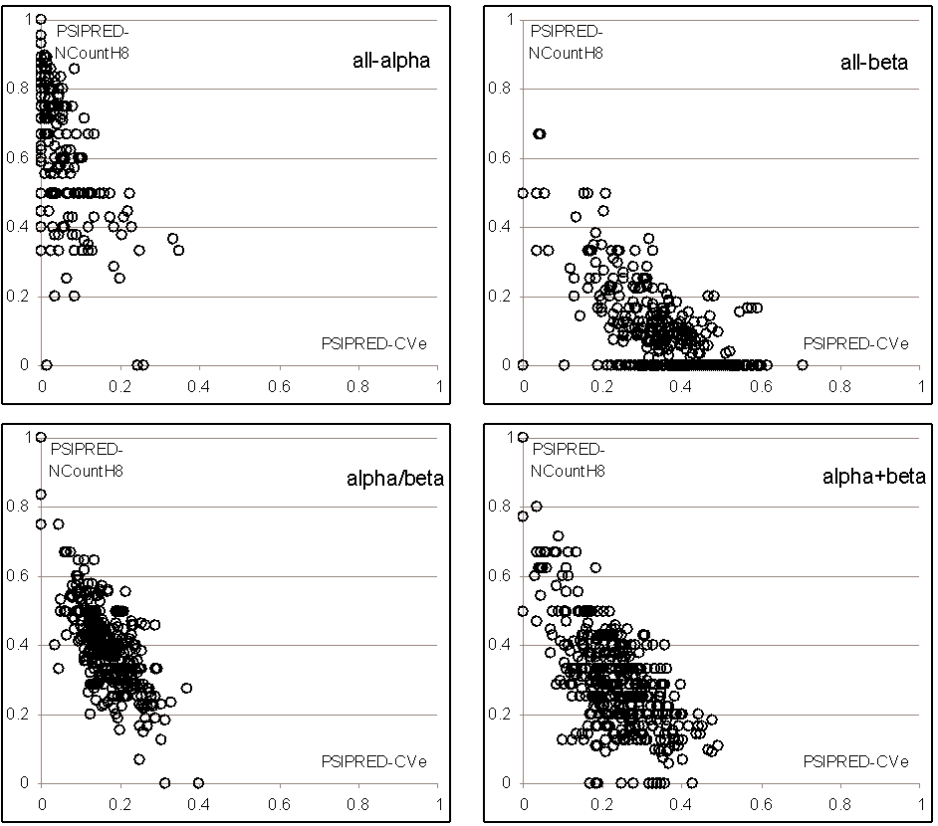
**Scatter plots of PSIPRED-*CV*_*E *_(x-axis) and PSIPRED-*NCount*_*H*_^8 ^(y-axis) features**. Top-left plot corresponds to sequences belonging to all-α class, top-right for all-β class, bottom-left for α/β, and bottom-right got α+β class.

Figure 1 shows scatter plots in which x-axis corresponds to PSIPRED-*CV*_*E *_and y-axis shows PSIPRED-*NCount*_*H*_^8^. The Figure shows that the values of the two features form relatively compact clusters for each of the structural classes. These clusters are also characterized by a small degree of spatial overlap, and thus the classifier can achieve good separation between all four structural classes. In other words, certain characteristics of the secondary structure that is predicted with PSI-PRED, which include composition, the count of secondary structure segments, and average/maximal size of the segments, provide information that differentiates between structural classes. For example, most proteins in all-α class include low number of residues that form β-strands (low value of PSIPRED-*CV*_*E*_) and high number of α-helix segments that are built of at least 8 AAs (high value of PSIPRED-*NCount*_*H*_^8^).

We also analyzed the *CV*_*L*---*G *_feature, which counts the number of occurrences of the LxxxG motif, where x is any AA. We found that higher number of these motifs in the sequences correlates with the α/β class. The corresponding minimal count of LxxxG motifs (count of all sequences that have at least that many motifs and belong to α/β class/count of all sequences that have at least that many motifs) in 25PDB dataset follows: 0 (346/1673), 1 (291/970), 2 (188/445), 3 (114/199), 4 (53/86), 5 (23/34), and 6 and higher (11/14). This shows that if a given sequence contains at least 5 LxxxG motifs, there is at least 68% probability that it belongs to α/β class. To show whether this motif is significant with respect to the structural class classification, we compute the expected number of motifs that are characterized by the same properties, i.e., they occur at least 5 times in at least 34 proteins and the corresponding probability of the most frequent class associated with that motif equals at least 68%, given that the structural classes are randomized. After randomly scrambling the class labels 10 times (we use the same proportion of class labels as in the original dataset), the expected value equals zero, and the average (over the 10 runs) highest probability obtained for such motifs equals 42.4. Given that the original class labels are used, two motifs that satisfy the above conditions are found, LxxxG and AxxL (with probability of 69% for α/β class), while their corresponding average (over the 10 runs) probabilities for the scrambled class labels equal 33.8 and 32.1, respectively. We note that although other, similar motifs (such as AxxL) that allow to differentiate between structural classes could be found (and were considered by our method), only LxxxG motif was found to be complementary to the remaining 8 features. A recent study that discusses other motifs that could be successfully used to address prediction of structural classes can be found in [29]. Analysis of the structures formed by the LxxxG motif reveals that many of them form either terminal end of an α-helix or a β-strand that folds into a parallel β-sheet. The two proteins that include the highest number of these motifs are (1) 1ofda2 domain that includes 9 motifs (6 form terminal end of an α-helix, 1 forms a parallel β-sheet and 2 form coils), and (2) 1r66 protein that includes 8 motifs (3 form terminal end of an α-helix, 2 form a parallel β-sheet and 3 form coils. This motif could serve as a signature for some proteins that belong to the α/β class.

### Application to fold classification

The SCPRED was coupled, as a post-processing filter, with two modern fold classification methods: PFP [50] and PFRES [51]. Fold classification aims at prediction of a fold for a given protein sequence, where multiple fold types are defined for each structural class. This means that each predicted fold can be automatically assigned to a corresponding structural class. Among the 27 folds predicted by PFP and PFRES, 6 (globin-like, cytochrome c, DNA/RNA-binding 3-helical bundle, four-helical up-and-down bundle, 4-helical cytokines, and EF Hand-like) belong to the all-α class, 9 (immunoglobulin-like beta-sandwich, cupredoxin-like, viral coat and capsid proteins, concanavalin A-like lectins/glucanases, SH3-like barrel, OB-fold, beta-Trefoil, trypsin-like serine proteases, and lipocalins) to the all-β class, 9 (TIM beta/alpha-barrel, FAD/NAD (P)-binding domain, flavodoxin-like, NAD (P)-binding Rossmann-fold domains, P-loop containing nucleoside triphosphate hydrolases, thioredoxin fold, ribonuclease H-like motif, alpha/beta-Hydrolases, and periplasmic binding protein-like I) to the α/β class, and 2 (beta-Grasp and Ferredoxin-like) to α+β class. The remaining fold concerns small proteins and thus was removed from our tests. The post processing was based on removing all predictions for which SCPRED and a given fold classification method predicted different structural classes, i.e., the predicted fold belonged to a different structural class than the class predicted with SCPRED. This approach is motivated by a hypothesis that if both methods provide consistent predictions (at the level of the structural classes) than the confidence in the fold prediction should be higher than in the case when the two methods provide different predictions. The accuracies of SCPRED, both fold classification methods and the coupled methods for the FC699 dataset (which was originally used to test both PFP and PRES), the sequences in FC699 that were kept (the same structural classes were predicted), and the removed sequences (different classes were predicted) are shown in Table 5.

**Table 5:** Comparisons of accuracies obtained by PFRES, PFP and coupled PFRES+SCPRED and PFP+SCPRED methods on FC699 dataset.

Entire FC699 dataset			Only kept sequences		Only removed sequences		
PFRES		SCPRED	PFRES + SCPRED				Coverage (% kept)

fold	class	class	fold	class	fold	class	
65.6	92.1	87.5	68.6	96.7	45.7	62.8	86.6%

PFP		SCPRED	PFP + SCPRED				Coverage (% kept)

fold	class	class	fold	class	fold	class	
30.9	65.8	87.5	47.3	97.0	3.8	14.1	62.4%

The "Entire FC699 dataset" column shows accuracies for PFRES, SCPRED and PFP methods for class/fold prediction on the FC699 dataset. The "Only kept sequences" column show accuracies obtained by the PFRES and PFP methods for sequences for which SCPRED predicted the same structural class as PFRES and PFP, respectively. The "Only removed sequences" column show accuracies obtained by the PFRES and PFP methods for sequences for which SCPRED predicted different structural class when compared with predictions of PFRES and PFP, respectively. The "Coverage" column shows the percentage of sequences for which the SCPRED and PFRES/PFP predicted the same structural class.

SCPRED obtains 87.5% accuracy on this dataset with sequences sharing pairwise twilight-zone similarity, which confirms high quality of our method. The PFRES and PFP method predict structural class with 92.1% and 65.8% accuracy, respectively. Although PFRES obtains higher accuracy than SCPRED, this method is more complex (uses 36 features and an ensemble classifier) and its predictions are complementary to the predictions of the SCPRED. Namely, the post-processing with SCPRED improved the structural class prediction accuracy by 4.6% and the fold classification accuracy by 3.1% as a trade-off for removing only 13.4% of the predictions. The structural class/fold prediction accuracy of the coupled method equals 68.6% and 96.7%, respectively. The removed sequences were characterized by much lower prediction quality, i.e., 45.7% for fold and 62.8% for the class predictions. When comparing the accuracies of the PFRES fold predictions before and after post-processing using predictions of SCPRED, the accuracies for 7 fold types were improved (the biggest 33.8% improvement was obtained for ferredoxin-like fold, and the second biggest, 8.6%, for SH3-like barrel fold), for 6 fold types they deteriorated (the biggest 19% loss was observed for ribonuclease H-like motif fold, and the second biggest, 16.3%, for FAD/NAD (P)-binding domain fold), and for the remaining 13 fold typed the accuracies did not change.

The improvements for the PFP method were more substantial. Post processing improved the fold prediction accuracy by 16.4% and the class prediction accuracy by 9.5% while removing 37.6% of predictions. The removed sequences were characterized by poorer predictions, i.e., 3.8% and 14.1% accuracies. Coupling of PFP with SCPRED as the post-processor resulted in improvements for 17 folds (the largest improvements of 66.6% and 46% were obtained for 4-helical cytokines and ferredoxin-like folds, respectively), deterioration of prediction accuracy for 1 fold (the loss of accuracy by 4% was observed for FAD/NAD (P)-binding domain fold), and accuracies for 8 folds did not change, when compared with accuracies obtained with standalone PFP.

The 5 folds for which post-processing with SCPRED improved accuracies in case of both PFP and PFRES include DNA/RNA-binding 3-helical bundle, immunoglobulin-like beta-sandwich, viral coat and capsid proteins, TIM beta/alpha-barrel, and ferredoxin-like, while the only fold that suffered consistent looses was FAD/NAD (P)-binding domain. In a nutshell, our tests show that coupling of the SCPRED with modern fold predictors provides improved accuracy and allows for removing poorer performing predictions.

## Conclusion

Prediction of structural classes for the "twilight-zone sequences", i.e., sequences that share twilight-zone similarity with sequences used for prediction, not only allows learning the overall folding type for a given protein sequence, but also helps in finding proteins that form similar folds in spite of low sequence similarity. Therefore, a high quality structural class predictor would be beneficial for in-silico prediction of tertiary structure of proteins with low sequence identity with respect to sequence used for prediction. To this end, we introduced the SCPRED method for prediction of structural classes from the "twilight-zone sequences". Our method provides predictions using SVM classifier and a compact feature vector to represent the input sequences. The features utilize information about secondary structure predicted with PSI-PRED and the protein sequence. Based on a comprehensive empirical comparison with other prediction methods on a set of over 1600 domains that share twilight-zone sequence similarity, the SCPRED is shown to obtain 80% accuracy, while the accuracies of other methods range between 35% and 63%. The main contribution of this paper is the new feature vector which was shown to uncover several relations between the predicted secondary structure and certain sequence motifs, and the structural classes. We show that the main source of the information that allows for successful predictions of structural classes is the secondary structure predicted with PSI-PRED. We also demonstrate that the proposed method can be successfully coupled with state-of-the-art fold classification methods to improve their accuracy. The empirical results show that although the proposed methods obtains favorable overall accuracy, the predictions for the mixed (α+β and α/β) classes are of lower quality when compared with the predictions for all-α and all-β classes. Therefore, investigations into improving predictions for the mixed classes would constitute an interesting subject for future work. One of such attempts could be implemented through designing of a classifier that better balances accuracies between all classes as a trade-of for lower overall accuracy.

## Methods

### Datasets

The SCPRED was tested on a large benchmark dataset, in which any pair of sequences shares twilight-zone similarity. This means that any test sequence shares twilight-zone identity with any sequence in the training set used to generate the proposed classification model. The dataset, referred to as *25PDB*, was selected using 25% PDBSELECT list [56], which includes proteins from PDB that were scanned with high resolution, and with low, on average about 25%, identity. The dataset was originally published in [26] and was used to benchmark two structural class prediction methods [27,29]. It contains 1673 proteins and domains, which include 443 all-α, 443 all-β, 346 α/β, and 441 α+β sequences.

A second dataset was used to verify whether the predicted structural classes can be used to perform post-processing of the results generated by two modern fold classification methods, PFP and PFRES. This dataset was originally introduced in [51] and includes sequences that belong to the 27 protein folds (as defined in SCOP) and that were deposited into PDB between 2002 and 2004. These sequences were filtered by CD-HIT [57] at 40% sequence identity. Next, the remaining sequences were aligned with the sequences from the 25PDB dataset and the sequences that were used to train PFP and PFRES methods using Smith-Waterman algorithm [58]. Only sequences that have less than 35% sequence identity with any sequence in 25PDB dataset and the two training sets were selected. We also removed proteins that belong to the "small protein" fold since they do not belong to any particular structural class, and they can be accurately classified based on the length of the underlying sequence. The final dataset, named *FC699*, includes 699 sequences that share low 40% identity with each other and 35% identity with sequences used to train SCPRED, PFP, and PFRES methods.

### Feature vector

The feature vector was derived from a comprehensive list of feature sets that were used for prediction of protein structural class, secondary structure content, function, family, structural flexibility, and solvent accessibility [23,26-29,33,59-72].

*Sequence length *denoted by *N*.

#### Index-based features

- The average isoelectric point $pI=\frac{1}{N}\sum_{i=1}^{N}pI_{i}$, where *pI*_*i *_is the pH at which a given amino acid type has a zero net charge; see Table 6 for *pI*_*i *_values.

- Auto-correlation functions $A_{n}^{a}=\frac{1}{N-n}\sum_{i=1}^{N-n}a_{i}a_{i+n}$ where *a *denotes an AA index. Two hydrophobicity indices, i.e., the Fauchere-Pliska's (*FH*) with *n *= 1,2,..,10 [73] and the Eisenberg's (*EH*) [74] with *n *= 1,2,..,6, the relative side-chain mass (*M*) [65] with *n *= 1,2,..,6, and the hydropathy (*Hp*) index [75] with *n *= 1,2..,9, were used, see Table 6.

**Table 6:** The values of AA indices that include average isoelectric point *pI*, Fauchere-Pliska's (*FH*) and the Eisenberg's (*EH*) hydrophobicity indices, and relative side-chain mass (*M*) and hydropathy (*Hp*) indices.

Name	Code	Index	Physicochemical index				
			
			*pI*	*FH*	*EH*	*Hp*	*M*
Alanine	A	1	6.01	0.42	0.62	1.8	0.115
Cysteine	C	2	5.07	1.34	0.29	2.5	0.777
Aspartate	D	3	2.77	-1.05	-0.9	-3.5	0.446
Glutamate	E	4	3.22	-0.87	-0.74	-3.5	0.446
Phenylalanine	F	5	5.48	2.44	1.19	2.8	0.36
Glycine	G	6	5.97	0	0.48	-0.4	0.55
Histidine	H	7	7.59	0.18	-0.4	-3.2	0.55
Isoleucine	I	8	6.02	2.46	1.38	4.5	0.00076
Lysine	K	9	9.74	-1.35	-1.5	-3.9	0.63
Leucine	L	10	5.98	2.32	1.06	3.8	0.13
Methionine	M	11	5.47	1.68	0.64	1.9	0.13
Asparagine	N	12	5.41	-0.82	-0.78	-3.5	0.48
Proline	P	13	6.48	0.98	0.12	-1.6	0.577
Glutamine	Q	14	5.65	-0.3	-0.85	-3.5	0.7
Arginine	R	15	10.76	-1.37	-2.53	-4.5	0.323
Serine	S	16	5.68	-0.05	-0.18	-0.8	0.238
Threonine	T	17	5.87	0.35	-0.05	-0.7	0.346
Valine	V	18	5.97	1.66	1.08	4.2	1
Tryptophan	W	19	5.89	3.07	0.81	-0.9	0.82
Tyrosine	Y	20	5.67	1.31	0.26	-1.3	0.33

- Cumulative auto-correlation functions

$$Acum_{n}^{a}=\frac{\sum_{i=1}^{N-n}\left(\sum_{j=1}^{i}a_{j}\right)\times \left(\sum_{j=1}^{i+n}a_{j}\right)}{N-n}\text{ where }a=\{FH\}\text{ with }n=1,2,..,6.$$

- Sum of hydrophobicities $H_{sum}^{a}=\sum_{i=1}^{N}a_{i}$ where *a *= {*FH*, *EH*}.

- Average of hydrophobicities $H_{avg}^{a}=\frac{\sum_{i=1}^{N}a_{i}}{N}$ where *a *= {*FH*, *EH*}.

- Sum of 3-running average of hydrophobicities

$$H_{sum3}^{a}=\sum_{i=1}^{N-3}(\sum_{j=i}^{i+3}a_{j})/3\text{ where }a=\{FH,EH\}.$$

#### AA composition-based features

- Composition vector *CV*_*i *_defined as the composition percentage of *i*^th ^AA in the sequence; see Table 6 for the AA index assignment.

- First and second order composition moment vector $CMV_{i}^{k}=\frac{\sum_{j=1}^{x_{i}}n_{ij}^{k}}{\prod_{d=1}^{k}\left(N-d\right)}$ where *n*_*ij *_represents the *j*^th ^position of the *i*^th ^AA and *k *= 1,2 is the order of the CMV. For *k *= 0 CMV reduces to CV.

- Count of collocated AA pairs *CV*_*AAiAAj*_, *CV*_*AAi*-*AAj*_, *CV*_*AAi*--*AAj*_, *CV*_*AAi*---*AAj*_, *CV*_*AAi*----*AAj *_where *i *and *j *denote two AAs and "-" denotes a gap. *AA*_*i*_*AA*_*j *_denotes dipeptides, *AA*_*i*_*-AA*_*j*_, denotes two AAs separated by a single gap, and *AA*_*i*_*--AA*_*j*_, *AA*_*i*_*---AA*_*j*_, and *AA*_*i*_*----AA*_*j *_denote two AAs separated by 2, 3 and 4 gaps, respectively. There are 400 pairs for each gap size.

#### Property group-based features

- R groups *RG*_*i *_where *i *= 1,2,...,5, *i *= 1 corresponds to nonpolar aliphatic AAs (AVLIMG), *i *= 2 to polar uncharged AAs (SPTCNQ), *i *= 3 to positively charged AAs (KHR), *i *= 4 to negative AAs (DE), and *i *= 5 to aromatic AAs (FYW).

- Electronic groups *EG*_*i *_where *i *= 1,2,...,5, *i *= 1 corresponds to electron donor AAs (DEPA), *i *= 2 to weak electron donor AAs (LIV), *i *= 3 to electron acceptor AAs (KNR), *i *= 4 to weak electron acceptor AAs (FYMTQ), and *i *= 5 to neutral AAs (GHWS).

- Chemical groups *CG*_*i*_, which are defined based on composition of chemical group that constitute the side chains where *i *= 1,2,...,10 corresponds to C, CAROM, CH, CH2, CH2RING, CH3, CHAROM, CO, NH, OH side chain groups, respectively.

- Exchange groups *XG*_*i *_where *i *= 1(HRK), 2(DENQ), 3(C), 4(STPAG), 5(MILV), 6(FYW), are supported by statistical studies and cluster AAs based on point mutation that represent conservative replacements through revolution.

- Hydrophobicity group *HG*_*i *_where *i *= 1,2 includes hydrophilic AAs (KHRDESTNQ), and hydrophobic AAs (VLIMAFPWYCG).

- Other groups *OG*_*i *_where *i *= 1,2,...,7 are defined based on molecular weights, i.e. tiny (AG), small (AGST) and bulky (FHWYR) AAs, and other groupings such as polar (DEKHRNTQSYW), aromatic (FHWY), charged (DEKHRVLI), and polar uncharged AAs (NQ).

- The composition percentage of each group was computed.

#### Predicted secondary structure content

- *contentH*_*f *_and *contentE*_*f *_where *H *corresponds to helix content, *E *corresponds to strand content and *f *denotes a prediction method were computed. The content quantifies the amount of residues in the sequence that assume helical and strand conformation. Based on [29], methods by Lin and Pan (*LP*) [65] and by Zhang and colleagues (*Z*) [76] were used to compute the content values using 10-fold cross validation on the 25PDB dataset.

#### Predicted secondary structure-based features

The SCPRED is the first structural class prediction method that uses the predicted secondary structure. We decided to use the PSI-PRED method [48,49] because it was recently shown to provide superior accuracy when compared with other state-of-the-art secondary structure prediction methods [41,77], and the YASPIN method which provides favorable accuracy for prediction of β-strands [41]. Although the secondary structure content reflects information about the secondary structure of the entire sequence, it does not provide information concerning individual secondary structure segments. Size (length) of secondary structure segments is one of the deciding factors when it comes to the assignment of the structural classes. We assert that in spite of the overall lower prediction accuracy when predicting sequences that share twilight-zone similarity with sequences used to perform prediction, the predicted secondary structure preserves enough information about the secondary structure segments to characterize the structural class. Our features do not use information about location of the segments in the sequence, because it might be of poor quality given the low sequence identity between our targets. Instead, we designed features that count the number of occurrences of distinct helix, strand and coil segments, and their average and maximal length. The 3-state predictions computed using the two prediction methods were used to generate the following features:

- Composition vector *CV*_*i *_for *i *= {*H*, *E*, *C*} and where *H *denotes α-helix, *E *denotes β-strand, and *C *denotes coil. *CV*_*H *_and *CV*_*E *_are equivalent to the secondary structure content.

- $CMV_{i}^{k}$ for *i *= {*H*, *E*, *C*} and *k *= 1,2,...,5.

- Normalized count of segments that include at least *k *residues

◦ for coil segments $NCount_{C}^{k}=\frac{\sum_{j=k}^{20}count_{C}^{j}}{\sum_{i=\{H,E,C\}}total_{i}}$ for *k *= 2,3,...0

◦ for α-helix segments $NCount_{H}^{k}=\frac{\sum_{j=k}^{20}count_{H}^{j}}{\sum_{i=\{H,E\}}total_{i}}$ for *k *= 3,4,...20

◦ for β-strand segments $NCount_{E}^{k}=\frac{\sum_{j=k}^{20}count_{E}^{j}}{\sum_{i=\{H,E\}}total_{i}}$ for *k *= 2,3,...20

where *count*_*C*, *E*, *H*_^*j *^denotes number of coil, β-strand, α-helix segments of length *j*, and *total*_*i *_denotes total number of all segments belonging to *i*^th ^secondary structure. The smallest α-helix segment is assumed to include at least 3 residues. The count of coil segments is normalized by the total number of all segment, while the counts of β-strand and α-helix segments are normalized by the total number of β-strand and α-helix segments. These differences in the normalizations accommodate for the all-α and all-β classes that may not include any β-strand and α-helix segments, respectively.

- Length of the longest segment *MaxSeg*_*i *_for *i *= {*H*, *E*, *C*}

- Normalized length of the longest segment *NMaxSeg*_i _= *MaxSeg*_*i*_/*N *for *i *= {*H*, *E*, *C*}

- Average length of the segment *AvgSeg*_*i *_for *i *= {*H*, *E*, *C*}

- Normalized average length of the segment *NAvgSeg*_i _= *AvgSeg*_*i*_/*N *for *i *= {*H*, *E*, *C*}

### Feature selection

The above features were processed by a feature selection method to obtain the input feature vector. The goal was to reduce the dimensionality and potentially improve the prediction accuracy when compared to using all features together. The selection was performed in two steps: (1) an automated feature selection method was applied to select a subset of the most promising features, and (2) the remaining features were processed manually to select the final subset of features. Since features selected in the first step may be correlated with each other or redundant, the second step aims at removing the overlapping features and selecting the minimal subset that still guarantees the same level of classification performance as the subset selected in the first step. Similarly to [29], the first step was implemented using a Correlation-based Feature Subset Selection method (CFSS) [78]. CFSS evaluates a given subset of features, which is found using best-first search based on hill-climbing with backtracking, by considering the individual predictive ability of each feature along with the degree of redundancy between them. Both steps were performed using 10-fold cross validation on the 25PDB dataset to avoid overfitting.

During the first step, only the features that were found significant by the CFSS in at least 5 folds were selected, see Table 7. In the second step, we attempted to remove each remaining feature and accepted such deletion if the corresponding accuracy of the structural class prediction (using 10 fold cross-validation on the 25PDB dataset with the SVM classifier as described in section "Classification Algorithm") was not lower then the accuracy when all 53 features selected in step 1 were used. The largest portion of the final set of selected features was computed from the secondary structure predicted with the PSI-PRED, namely, 8 out of 9 (see Table 7). They include four features that were computed based on α-helix segments: PSIPRED-*NCount*_*H*_^6^, PSIPRED-*NCount*_*H*_^8^, PSIPRED-*CMV*_*H*_^1^, PSIPRED-*NAvgSeg*_*H*_, and another four that were based on β-strand segments: PSIPRED-*CV*_*E*_, PSIPRED-*NCount*_*E*_^5^, PSIPRED-*MaxSeg*_*E*_, and PSIPRED-*NAvgSeg*_*E*_. The remaining attribute is based on count of a collocated *CV*_*L*---*G *_pair, which is consistent with our prior results [29]. Table 7 shows that step 1 of our feature selection resulted in improved prediction accuracy, while step 2 provided further reduction in the dimensionality while preserving the accuracy.

**Table 7:** Summary of the feature selection results.

Feature set		# features		
		
		all	after step 1	after step 2
Length		1	0	0
Index-based		50	5	0
CV and CMV		60	2	0
CV for collocated AAs		2000	4	1
Property group-based		35	1	0
Predicted secondary structure content		4	2	0
Predicted secondary structure-based	with PSI-PRED	86	27	8
	with YASPIN	86	12	0

Total # of features		2322	53	9

10 fold cross validation accuracy for prediction on 25PDB dataset		73.2%	80.2%	80.1%

The "feature set" columns defines categories of the considered features, the "all" column shows the total number of features in a given category, while the "after step 1" and "after step 2" columns show the corresponding number of features from a given category that were selected in the step 1 and step 2 of the feature selection procedure, respectively.

To demonstrate the importance of the features computed from the predicted secondary structure, and especially those based on predictions coming from PSI-PRED, we performed the same feature selection but when considering only 86 features from the PSI-PRED predicted secondary structure. After step 1, 28 features were selected and the corresponding 10 fold cross-validation accuracy on 25PDB dataset was 79.9%. After step 2, we further reduced the number of features to 13 with the corresponding accuracy of 79.7%.

### Classification algorithm

We use support vector machine (SVM) classifier [79] that was previously applied for structural class prediction [27,34-36]. Given a training set of data point pairs (*x*_*i*_, *c*_*i*_), *i *= 1, 2, ... *n*, where *x*_*i *_denotes the feature vector, *c*_*i *_= {-1, 1} denotes binary class label, *n *is the number of training data points, finding the optimal SVM is achieved by solving:

$$\begin{array}{c} \min{\left\|w\right\|}^{2}+C\sum_{i}\xi_{i}\\ \text{such that }c_{i}(wz_{i}-b)\geq 1-\xi_{i}\text{ and }1\leq i\leq n \end{array}$$

where *w *is a vector perpendicular to *wx*-*b *= 0 hyperplane that separates the two classes, *C *is a user defined complexity constant, *ξ*_*i *_are slack variables that measure the degree of misclassification of *x*_*i *_for a given hyperplane, *b *is an offset that defines the size of a margin that separates the two classes, and *z *= φ(*x*) where *k*(*x*, *x*') = φ(*x*)·φ(*x*') is a user defined kernel function.

The SVM classifier was trained using Platt's sequential minimal optimization algorithm [80] that was further optimized by Keerthi and colleagues [81]. The structural class prediction that includes multiple classes is solved using pairwise binary classification, namely, a separate classifier is build for each pair of classes. Two popular families of kernel functions including polynomials and radial basis functions (RBF) were used. The kernel function selection and parameterization as well as selection of the complexity constant value were performed based on 10-fold cross validation on the 25PDB dataset using 53 features selected in step 1 of the feature selection procedure. The final classifier uses *C *= 2 and the RBF kernel

k(xi,xi')=e−γ‖x−x'‖2 where γ=0.6

The classification algorithms used to develop and compare the proposed method were implemented in Weka [82]. We note that computation of the SVM model using the 25PDB dataset encoded using the selected 9 features takes less than 2 seconds on a desktop computer equipped with Pentium processor at 2.8GHz.

## Availability & requirements

The prediction model and datasets can be freely accessed at 

## Authors' contributions

LK contributed to the conception and design of the prediction method, prepared the datasets, designed and computed the features, performed feature selection and experimental comparison, and helped with evaluation of the results. KC contributed to the conception of the prediction method and helped with evaluation of the results. KC prepared the datasets, designed and computed the features, and helped with experimental comparison. All authors have drafted, corrected and approved the manuscript.
